# Adjuvant Treatment Recommendations in Early-Stage Endometrial Cancer: What Changes With the Introduction of The Integrated Molecular-Based Risk Assessment

**DOI:** 10.3389/fonc.2021.612450

**Published:** 2021-09-01

**Authors:** Camilla Nero, Francesca Ciccarone, Antonella Pietragalla, Simona Duranti, Gennaro Daniele, Giovanni Scambia, Domenica Lorusso

**Affiliations:** ^1^Direzione Scientifica, Fondazione Policlinico Universitario A. Gemelli IRCCS, Rome, Italy; ^2^Dipartimento di Scienze della vita e sanità pubblica, Università Cattolica del Sacro Cuore, Rome, Italy

**Keywords:** endometrial cancer, molecular classification, adjuvant treatment, recommendations, risk factors

## Abstract

Adjuvant therapy recommendations for endometrial cancer were historically based on the individual patient’s risk of disease recurrence using clinicopathologic factors such as age, stage, histologic subtype, tumor grade, and lymphovascular space invasion. Despite the excellent prognosis for early stages, considerable under- and overtreatment remains. Integrated genomic characterization by the Cancer Genome Atlas (TCGA) in 2013 defined four distinct endometrial cancer subgroups (POLE mutated, microsatellite instability, low copy number, and high copy number) with possible prognostic value. The validation of surrogate markers (p53, Mismatch repair deficiency, and POLE) to determine these subgroups and the addition of other molecular prognosticators (CTNNB1, L1CAM) resulted in a practical and clinically useful molecular classification tool. The incorporation of such molecular alterations into established clinicopathologic risk factors resulted in a refined, improved risk assessment. Thus, the ESGO/ESTRO/ESP consensus in 2020 defined for the first time different prognostic risk groups integrating molecular markers. Finally, the feasibility and clinical utility of molecular profiling for tailoring adjuvant therapy in the high-intermediate-risk group is currently under investigation (NCT03469674).

## Overview

Endometrial cancer (EC) is the most common gynecological cancer in developed countries; the majority of cases are diagnosed at an early stage and addressed to surgical treatment (1). Traditionally, ECs have been categorized into two pathogenetic types based on clinical, metabolic, and endocrine characteristics: type I tumors (60–70%), associated with estrogen excess, obesity, hormone-receptor positivity, and endometrial hyperplasia, with favorable outcomes, and type II tumors (30–40%), more common in non-obese women, associated with an atrophic endometrium, with aggressive clinical behavior and poor outcome (2).

Adjuvant therapy recommendations have traditionally been based on the individual patient’s risk of disease recurrence using clinicopathologic factors such as age, stage, histologic subtype, tumor grade, and lymphovascular space invasion (LVSI) (3, 4). In particular, the ESMO/ESGO/ESTRO (European Society for Medical Oncology–European Society for Radiotherapy and Oncology–European Society of Gynaecological Oncology) consensus in 2016 proposed five risk groups to guide adjuvant therapy use (low, intermediate, high-intermediate, high, advanced/metastatic) (4).

Overall, risk-adapted treatments achieve excellent prognosis for early-stage type I ECs, with 10-year overall survival exceeding 80% (5, 6).

However, a small but substantial number of patients with favorable prognostic background unexpectedly experience recurrence of disease and poor survival (5–8), and it has been calculated that up to 10% of them will experience distant metastasis (7).

On the other hand, a not-negligible number of patients with unfavorable prognostic factors that are usually treated will never experience recurrence: in particular, seven high-intermediate-risk patients need to undergo vaginal brachytherapy (EBRT) to prevent one recurrence (7).

In 2013, the Cancer Genome Atlas (TCGA) defined four distinct EC subgroups (POLE mutated, microsatellite instability, low copy number, and high copy number) with possible prognostic value, and many others confirmed these data in external cohorts (3–5, 7, 8). Molecular risk classes are not completely superimposable with clinicopathological categories, but the combination of both models has been shown to perform better in terms of prognosis than the single ones by themselves (7).

The most recent ESGO/ESTRO and the European Society of Pathology (ESP) ESP recommendations, published at the end of 2020, incorporated molecular and clinicopathological features in an integrated classification system in order to guide adjuvant treatment choices (9).

## A Comprehensive Genomic and Transcriptomic Analysis Through Next-Generation Sequencing (NGS): A Genomic Classification

In 2013, the Cancer Genome Atlas Research Network (TCGA) has reported a comprehensive genomic and transcriptomic analysis of 373 EC cases, mainly endometrioid (307) (8).

**Figure 1:**
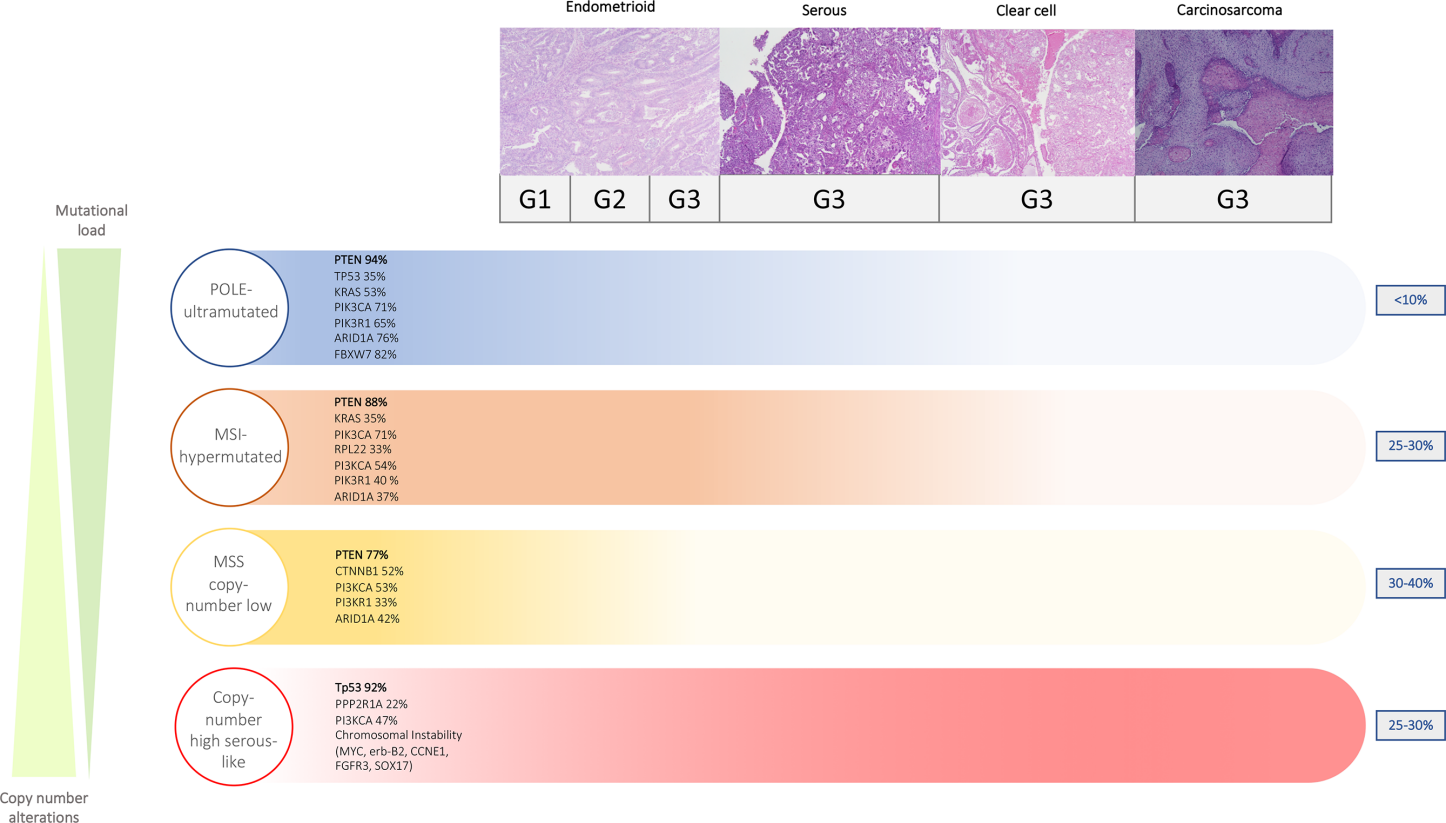
Distribution of molecular features across different EC histotypes.

This characterization categorized EC tumors into four genomic classes with different molecular and prognostic profiles. Such molecular analyses were proven feasible (>96%) and highly reproducible in external cohorts of patients (7, 10, 11). The distribution of histologic subtypes into the four molecular classes is reported in **Figure 1**. 

### POLEmut

This molecular class is defined by pathogenic mutations in the exonuclease domain of DNA polymerase epsilon (10).

This gene encodes a catalytic subunit of DNA polymerase epsilon involved in nuclear DNA replication and repair. The most common alterations in POLE detected in EC samples are hotspot mutations at P286R, V411L, S297F, A456P, and S459F (10, 12–14).

Overall, this genomic class is associated with excellent prognosis. It accounts for less than 10% of all EC cases, and it is associated with low copy-number aberrations and a very high mutational burden (232 × 10^−6^ mutations/Mb).

The associated morphological characteristics of this subgroup include high rate of tumor-infiltrating lymphocytes and/or peritumoral lymphocytes, morphologic heterogeneity/ambiguity, bizarre/giant tumor cell nuclei, endometrioid histotype but also clear cell carcinomas, undifferentiated carcinomas, and carcinosarcomas (13, 15).

Approximately 65% of this molecular class is associated with intermediate and high-risk phenotype according to ESMO 2013 classification (16). In particular, it is frequently associated with grade 3 endometrioid cancers. Given the favorable prognosis of this subgroup, no adjuvant treatment could be suggested, reducing the possible overtreatment, particularly in the high-intermediate- and high-risk group. The PORTEC-4a trial (ISRCTN11659025) will answer whether this strategy is safe and efficient in the high-intermediate-risk subgroup (17).

### Microsatellite Unstable (MSI Hypermutated)

This molecular class is characterized by the presence of microsatellite instability.

MSI represents the phenotypic evidence that DNA mismatch repair (MMR) is not functioning normally. The MMR is a system for recognizing and repairing erroneous insertion, deletion, and misincorporation of bases that can arise during DNA replication and recombination, as well as repairing other forms of DNA damage.

The most common alteration in MMR detected in EC samples is MLH1 promoter methylation (8).

Overall, this genomic class is associated with intermediate prognosis.

It encounters for 25–30% of all EC cases and is characterized by low copy-number aberrations and high mutational burden (18 × 10^−6^ mutations/Mb).

The associated morphological characteristics of this class include lower uterine segment location, mainly endometrioid histology, mucinous differentiation, tumor-infiltrating lymphocytes, peritumoral lymphocytes, and with lymphovascular space invasion, mainly substantial (18–20).

Approximately 30% of this molecular class is associated with the low-risk phenotype according to ESMO 2013 classification (16).

Around 10–14% of EC MMRd patients are estimated to have a Lynch syndrome. In particular, chances are higher in case of MSH2−/MSH6− or PMS2− and lower in case of MLH1− (40 and 67% *vs* 2%) (21, 22).

### Copy-Number High (Serous-Like)

This genomic class is defined mainly by TP53 mutations (8).

P53 gene encodes for p53 protein (TP53) mainly involved in cell cycle arrest, DNA repair, senescence, apoptosis induction, but also cell metabolism regulation and cell response to oxidative stress (23).

Overall, this genomic class is associated with unfavorable prognosis.

It encounters for 25–30% of all EC cases and is characterized by a low mutation rate (2·3 × 10^−6^ mutations/Mb) and high copy-number aberrations.

The associated morphological characteristics of this class include serous, endometrioid, and mixed serous and endometrioid histology, grade 3, poor inflammatory stroma (23).

Approximately 25% of this molecular class is associated with low- and intermediate-risk phenotypes according to ESMO 2013 classification (16). About 4% of EC are classified as multiple classifier at molecular profiling, and when POLE and p53abn coexist, the prognosis is driven by POLE. In the same way, when MMR-d and p53abn coexist, the prognosis is driven by MMR-d (24).

### Copy-Number Low

This genomic class comprises mainly microsatellite-stable cancers characterized by frequent CTNNB1 mutations (8).

Overall, this genomic class is associated with intermediate prognosis.

It encounters for 30–40% of all EC cases and is characterized by a low mutational burden (2.9 × 10^−6^ mutations/Mb) and low copy-number aberrations.

The associated morphological characteristics of this class include endometrioid histology, grade 1–2, very poor inflammatory stroma (25).

Approximately 50% of this molecular class is associated with the low-risk phenotype according to ESMO 2013 classification (16).

## From NGS to Immunohistochemistry (IHC): The Molecular EC Classification

Methodologies used for the TCGA study are costly, complex, and unsuitable for wider implementation in clinical practice. In 2015, a pragmatic molecular classifier based on surrogate immunohistochemistry assays was developed and validated in internal and external cohorts (10, 11, 26, 27). It was aimed at replicating and replacing the TCGA classification, which relied on whole-exome sequencing (WES). This approach was tested by multiple study groups, which makes evidences concerning its feasibility and reliability especially robust (28).

The “MSI hypermutated” group was identified as MMR deficient (MMRd) using MMR IHC testing (MLH1, MSH2, MSH6, and PMS2), and it showed high concordance with MSI assay by NGS.

The “high copy number” group was identified as p53-abnormal (p53abn) determining p53 status by IHC testing; the subgroup obtained, however, was not completely equivalent to the TCGA one.

No surrogate was found for POLEmut detection; thus, NGS was maintained.

The “low copy number” group was determined by exclusion and called non-specific molecular profile (NSMP).

## Integrated Clinicopathologic and Molecular Classification: The ESGO/ESTRO/ESP 2020 Risk Classification and Adjuvant Treatment Recommendations

The integration of molecular and clinicopathological factors in early-stage ECs in various PORTEC trials cohorts resulted in a stronger model with improved risk prognostication (7).

In particular, the AUC of the integrated molecular risk assessment showed a substantial improvement in predicting locoregional recurrence, distant recurrence, and overall survival compared to clinicopathological classification alone.

Its main implication is to guide clinicians’ choices in terms of fertility-sparing treatments, surgery, adjuvant therapy, and surveillance in order to improve outcomes for women with EC.

In the light of available evidences, the ESGO/ESTRO/ESP decided to jointly update EC management evidence-based guidelines, implementing the use of molecular classification.

Risk group classification includes both cases that undergo molecular profiling and cases who did not. If molecular classification tools are not available, traditional pathologic features are used to classify EC patients. The main characteristics of the large trials included in the consensus, which guided treatment decision making, are summarized in **Table 1**.

**Table 1:** Relevant clinical trials for the ESGO/ESTRO/ESP consensus.

Clinical trial	Reference	Years	Numberof patients enrolled	Inclusion criteria	Study design	Treatments	Conclusions	Note
**PORTEC-1**	Creutzberg et al. (29) Lancet	1990–1997	714	• Stage IC grade 1–2 • Stage IB Grade 2–3 • Endometrial adenocarcinoma	RCT 1:1	EBRT (46 Gy using 2 Gy daily fractions) *vs* NAT	EBRT significantly reduced the risk of locoregional recurrence, without survival benefit.	• Routine lymphadenectomy not performed
**PORTEC-2**	Nout et al. (30) JCO	2002–2006	427	• Age >60, stage 1 grade 1–2 • Age >60, stage 1 grade 3 • Any age and stage 2A grade 1–2 or grade 3 with <50% invasion	RCT 1:1	Pelvic EBRT (46 Gy in 23 fractions) *vs* VBT (21 Gy HDR in 3 fractions, or 30 Gy LDR)	VBT is effective in preventing vaginal recurrence.	• Routine lymphadenectomy not performed
**PORTEC-3**	De Boer et al. (31) Lancet	2006–2013	660	• Stage 1A endometrioid grade 3, LVSI+ • Stage IB endometrioid grade 3 • Stage II endometrioid • Stage IIIA, IIIB IIIC endometrioid Serous EC with invasion), IB, II, or III. • Clear-cell EC with stages IA (with invasion), IB, II, or III. stages IA (with	RCT 1:1	EBRT (48·6 Gy in 1,8 Gy fractions given on 5 days per week) *vs* radiotherapy and chemotherapy (consisting of two cycles of cisplatin 50 mg/m2 given during radiotherapy, followed by four cycles of carboplatin AUC5 and paclitaxel 175 mg/m^2^)	EBRT+CHT for high-risk endometrial cancer did not significantly improve overall survival but improved 5-year failure-free survival compared with EBRT alone.	• Routine lymphadenectomy not performed
**GOG-99**	Keys et al. (32) Gyn Oncol	1987–1995	392	• IB • IC • IIA (occult) • IIB [occult]	RCT 1:1	EBRT 50.40 Gy given more than 28 fractions of 180 cGy *vs* NAT	EBRT decreases the risk of recurrence, but should be limited to high-intermediate-risk patients.	cycles of carboplatin AUC5 and paclitaxel 175 mg/m^2^)Selective bilateral pelvic, and para-aortic lymphadenectomy
**ASTEC/EN5**	ASTEC/EN.5 Study Group, Lancet 2009	1996–2005	905	• FIGO stage IA G3 • IB grade 3 • IC all grades • Papillary serous all stages and grades • Clear-cell histology all stages and grades	RCT 1:1	EBRT (40– 46 Gy in 20–25 daily fractions) *vs* NAT	EBRT did not improve overall survival compared to observation.	• Lymphadenectomy as part of surgical staging was not a requirement

RCT, randomized control trials; EBRT, external beam radiotherapy; VBT, vaginal brachytherapy; CHT, chemotherapy; FIGO, International Federation of Gynecology and Obstetrics 1999; NAT, non-adjuvant treatment.

Clinicopathological factors include the following:

- age
- International Federation of Gynecology and Obstetrics (FIGO) stage 2009
- depth of myometrial invasion
- tumor differentiation grade
- tumor type (endometrioid *vs* non-endometrioid)
- lymphovascular space involvement (LVSI)
Molecular features include the following:
- POLE mutation analysis by DNA sequencing
- p53 assessed by IHC
- MLH1, MSH2, MSH6, and PMS2 assessed by IHC

A consensus definition for LVSI in the literature is lacking. It reported good inter-observer agreement in discriminating “true LVSI” from “LVSI mimics” and in grading the extent of LVSI through a semiquantitative system (33). Nevertheless, some problematic cases exist. In addition, substantial LVSI in EC seems to have a stronger prognostic significance than focal LVSI (34–36).

Overall, the new ESTRO/ESGO/ESP guidelines published in 2020 integrate molecular into clinical classification and encouraged molecular classification in all EC especially high-grade tumors with only POLE mutation analysis possibly omitted in low-risk and intermediate-risk carcinoma with low-grade histology. Based on this, p53abn tumors with myometrial invasion are considered and treated as high-risk patients with chemotherapy or the combination of chemotherapy and radiotherapy. Stage I–II POLEmut ECs without residual disease are considered low-risk patients for which no adjuvant treatment is recommended.

### Low-Risk Class

This risk class includes patients with one of the following conditions:

- FIGO 2009 stage IA (<50% myometrial invasion), endometrioid histology, grade 1, LVSI negative
- POLEmut in FIGO 2009 stage I–II EC without residual disease
- MMRd/NSMP in FIGO 2009 stage IA G1, LVSI negative or focal

Routine lymphadenectomy for nodal staging purposes is generally not recommended for this group (37, 38). Sentinel lymph node biopsy can be considered for staging purposes, but it can be omitted in cases without myometrial invasion (38, 39). The incidence of recurrence after surgery alone is <5% (40). No adjuvant treatment is recommended for this group.

### Intermediate-Risk Class

This risk class includes patients with one of the following conditions:

- FIGO 2009 stage IB (<50% myometrial invasion), endometrioid histology, grade 1–2, LVSI negative or focal
- FIGO 2009 Stage IA endometrioid, grade 3, LVSI negative or focal
- FIGO 2009 Stage IA non-endometrioid (serous, clear cell, undifferentiated carcinoma, carcinosarcoma, mixed) without myometrial invasion
- MMRd/NSMP in FIGO 2009 stage IB, endometrioid histology, grade 1–2, LVSI negative or focal
- MMRd/NSMP in FIGO 2009 stage IA, endometrioid, G3, + high-grade, LVSI negative or focal
- p53abn in FIGO 2009 stage IA without myometrial invasion

Lymphadenectomy can be considered as a staging procedure to better tailor adjuvant treatment (37, 41).

The incidence of recurrence after surgery alone is between 5 and 10% (29, 32, 42, 43).

EBRT is recommended to decrease vaginal recurrence since it has been shown to reduce the risk of local relapse (29, 32, 42, 43). Observation is an option, especially for patients aged <60 years (44).

For p53abn FIGO 2009 stage IA without myometrial invasion cases, adjuvant treatment should be discussed on a case-by-case basis since specific data are missing.

### High-Intermediate-Risk Class

This risk class includes patients with one of the following conditions:

- FIGO 2009 stage IA, regardless of grade or depth of invasion with LVSI unequivocally positive
- FIGO 2009 stage IB, grade 3, regardless of LVSI status
- FIGO 2009 Stage II

Lymphadenectomy for nodal staging purposes can be considered (45).

The incidence of recurrence after surgery alone is between 12 and 14% (29, 32).

For those patients who underwent surgical nodal staging documenting negative nodes, VBRT is recommended to decrease vaginal recurrence, but no adjuvant therapy with close follow-up is an alternative acceptable option (4). In the case of substantial LVSI, EBRT can be considered in order to reduce the risk of pelvic and para-aortic nodal relapse (46). Similarly, cases displaying grade 3 tumors and/or substantial LVSI could benefit from adjuvant chemotherapy (31).

In patients for which lymph nodal status is unknown, VBRT is recommended for those patients who have LVSI negative, while EBRT is recommended for LVSI unequivocally positive to decrease pelvic recurrence (31, 46). Systemic therapy is considered of uncertain benefit (31).

### High-Risk Class

This risk class includes patients with the following characteristics:

- FIGO 2009 stage I non-endometrioid (serous, clear cell, undifferentiated carcinoma, carcinosarcoma, mixed) with myometrial invasion, and with no residual disease
- FIGO 2009 stage I p53abn endometrial carcinoma with myometrial invasion, with no residual disease.

There is no agreement on the role of lymphadenectomy in this risk class (4).

The 5-year incidence of recurrence (vaginal, pelvic, or distant) is around 41% (29, 31, 46).

For this class, EBRT with concurrent and adjuvant chemotherapy or alternatively sequential chemotherapy and radiotherapy is recommended (29, 31, 46–48). However, chemotherapy additional benefit is unclear for patients with clear-cell carcinomas. Chemotherapy alone can be an alternative option (49).

## Additional Features

Additional prognostic risk factors such as L1CAM and mutations in exon 3 of CTNNB1 later emerged and demonstrated to better mark differences in terms of prognosis among the four classes (16, 34, 50–52). Overall, three different prognostic profiles were delineated (see **Table 2**).

**Table 2:** Three different prognostic profiles in FIGO 2009 Stage I EC, delineated including additional molecular factors.

	FAVORABLE	INTERMEDIATE	UNFAVORABLE
**Characteristics**	POLE mut OR NSMP CTNNB1 WT	NSMP CTNNB1 mut MMRd	P53abn LVSI substantial >10% L1CAM

These additional features and, as a consequence, these profiles were not included in the most recent guidelines but were adopted in PORTEC-4a study to assign adjuvant treatment in the experimental arm (17).

### CTNNB1

CTNNB1 gene encodes β-catenin protein, involved in regulation and coordination of cell adhesion and cell signaling.

In particular, within the copy number low group, CTNNB1 exon 3 mutation status was found prognostic for distant recurrence in EC (7).

Although nuclear expression of β-catenin could be an IHC surrogate of CTNNB1 exon 3 mutations, NGS testing remains the gold standard (52–55).

CTNNB1 status helped distinguishing, within this class, a favorable group (CTNNB1-wild type) with a similar prognosis to POLEmut tumors, from an unfavorable group (CTNNB1-mutant), with a similar prognosis to MMRd.

### L1CAM

L1CAM is a 200 to 220 kDa membrane glycoprotein of the immunoglobulin superfamily and is crucially involved in processes of neurogenesis (56).

The established ≥10% threshold for positivity was based on the cutoff that best correlated with prognosis (57). It has been shown that patients bearing L1CAM-positive cancers have poorer disease-free and overall survival (51).

L1CAM positivity was mainly, but not exclusively, found in intermediate- and high-risk cancers (13.2 *vs* 25.8% in low and intermediate, respectively) (51). Moreover, it was associated with histopathological high grade and increasing depth of myometrium infiltration (58).

Given the association with an overwhelming increase in the likelihood of distal or local recurrence and poor overall survival, its presence indicates the need for adjuvant treatment (51, 59).

## Conclusions

The traditional dualistic histopathologic classification that split EC into two groups, type I and type II cancer, is not more adherent to practical necessity of the clinicians. In recent years it has become increasingly clear that the traditional classification lacks reproducibility and yields heterogeneous molecular groups, hampering advances and implementation of precision medicine. This is particularly problematic for future clinical trials with targeted approaches that will demand inclusion of cancers with molecular similarities. The endometrial cancer classification proposed by TCGA would serve this purpose well, as it is based upon the combination of somatic mutational burden and somatic copy number alterations. Moreover, several publications on large and clinically well annotated (trial) cohorts have shown that surrogate IHC markers can be utilized for a TCGA-inspired molecular classification in routine surgical pathology, without the need for extensive sequencing. These surrogate markers have been extensively studied and show good performance. The prognostic value has been well established, with POLEmut EC having an excellent outcome and p53abn EC having the poorest clinical outcome, independent of risk group, type of adjuvant treatment, tumor type, or grade. This implies that de-escalation of adjuvant treatment for POLEmut EC patients should be explored, as is currently being done in the clinical PORTEC4a trial. Furthermore, recent data strongly suggest that the benefit for the addition of chemotherapy in the adjuvant treatment is limited to p53mut EC, which includes most serous cancers but also a significant portion of other histologic subtypes such as carcinosarcomas, thus suggesting an escalation of adjuvant treatment with chemotherapy combined with radiation when p53 mutation is detected. The implementation of molecular classification into clinical classification has the potential to serve in improving patient management by reducing over- and undertreatment (60). The use of this novel classification in routine clinical practice and future trial designs should be encouraged. Currently, one trial (PORTEC-4a) is ongoing to determine whether adjuvant treatment can be based on a molecular-integrated risk profile rather than standard clinicopathological risk factors in high-intermediate-risk EC patients. The preliminary report of the first 50 patients enrolled showed that molecular assessment is feasible, but patients’ acceptance rate was not completely satisfactory (around 35%) (17).

Nevertheless, possible technical limits such as the need of assay harmonization as well as the lengthening of reporting times should be addressed.

## Author Contributions

CN: Conceptualization; Metholodology; Data curation; Roles/Writing - original draft; FC: Data curation; AP: Data curation; SD: Data curation; GD: Supervision; Writing - review & editing. GS: Supervision; Writing - review & editing. DL: Funding acquisition; Supervision; Writing - review & editing. All authors contributed to the article and approved the submitted version.

## Conflict of Interest

The authors declare that the research was conducted in the absence of any commercial or financial relationships that could be construed as a potential conflict of interest.

## Publisher’s Note

All claims expressed in this article are solely those of the authors and do not necessarily represent those of their affiliated organizations, or those of the publisher, the editors and the reviewers. Any product that may be evaluated in this article, or claim that may be made by its manufacturer, is not guaranteed or endorsed by the publisher.
